# Electrochemical Impedance Spectroscopy for the Dielectric Characterization of Crude Oils and Their Maltene Fractions

**DOI:** 10.1021/acsomega.6c04438

**Published:** 2026-06-12

**Authors:** Moisés O. dos Anjos, Eliane S. de Souza, Tiago L. da Silva, Rodrigo de S. Melo

**Affiliations:** 1 Electroanalytics and Advanced Materials Group, Multidisciplinary Institute of Chemistry, Multidisciplinary Center UFRJ-Macaé, Federal University of Rio de Janeiro, Avenida Aluízio da Silva Gomes, 50, Bairro da Glória, Macaé, Rio de Janeiro 27930-560, Brazil; 2 Laboratory of Petroleum Engineering and Exploration (LENEP), North Fluminense State University (UENF), Rodovia Amaral Peixoto, Km 163, Bairro Imboassica, Macaé, Rio de Janeiro 27925-310, Brazil

## Abstract

The dielectric characterization of crude oils and their fractions, particularly asphaltenes and maltenes (saturated and aromatic hydrocarbons and resins) are essential for understanding critical phenomena in the petroleum industry, such as oil aggregation, colloidal stability, and electrical conductivity. This work advances the dielectric characterization of crude oils and their fractions using electrochemical impedance spectroscopy over a wide frequency range, combining equivalent-circuit analysis with rigorous model validation. Measurements were performed on two crude oil samples (A and M) from the same source rock but from different reservoir rocks and their corresponding maltene fractions at 25 ± 0.1 °C using an electrochemical cell with carbon-steel electrodes separated by a 5 mm gap. Impedance spectra were acquired over the frequency range of 10 kHz to 10 mHz with a logarithmic distribution of 10 points per decade using an AC perturbation amplitude of 200 mV. Nyquist diagrams exhibit single semicircular arcs for all samples, a characteristic feature of highly resistive dielectric systems. Quantitative analysis through equivalent circuits revealed a clear resistivity hierarchy: Rct (A Oil) < Rct (A Maltene) < Rct (M Oil) < Rct (M Maltene), indicating that the presence of resins in the M Maltene and asphaltenes + resins in the M Oil strongly influences dielectric behavior. Samples from M Oil exhibit significantly higher charge-transfer resistance, suggesting that M Oil is denser and more concentrated in polar organic compounds (resins and asphaltenes), making it structurally more complex. Asphaltene removal results in a substantial increase in the insulating character of the maltene matrix, demonstrating that asphaltenes act as charge-active centers, facilitating conduction through mechanisms such as charge hopping. The results reinforce the importance of asphaltenes in modulating charge-transport properties and dielectric stability in petroleum systems, with significant implications for industrial applications related to stability, aggregation, and transport of oils.

## Graphical abstract

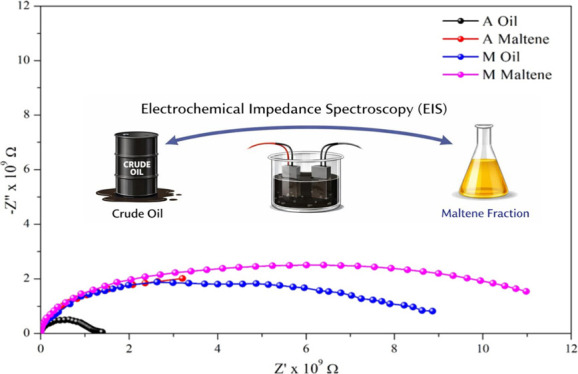

## 1 Introduction

Petroleum is a mixture of saturated and aromatic hydrocarbons, and polar organic compounds such as resins and asphaltenes, which contain heteroatoms such as of nitrogen, oxygen, and sulfur. Petroleum also contains metals such as nickel and vanadium, associated with structures like porphyrins. Asphaltenes have a complex molecular structure, composed of aromatic rings with alkyl side chains, and their interaction with the maltene fractions (saturated and aromatic hydrocarbons, and resins), directly influences their stability in the oil.^1,2^

The dielectric characterization of crude oils and their fractions, particularly asphaltenes and maltenes, is essential for understanding critical phenomena in the petroleum industry, such as oil aggregation, colloidal stability, and electrical conductivity. Recently, Electrochemical Impedance Spectroscopy (EIS) has been employed to investigate these phenomena in a nondestructive manner and with high sensitivity.^3^

The polarity of asphaltenes, their tendency to aggregate, and the formation of nanostructures represent major challenges in the physics of petroleum resources. A recent study associated asphaltene polarity with their ability to cluster using dielectric relaxation spectroscopy, showing that more polar molecules tend to form larger and more stable aggregates at high concentrations.^4^ These nanoaggregates directly modify the relaxation processes, which is reflected in the characteristic frequencies observed in impedance spectra. Moreover, the influence of electric fields on asphaltenes has been extensively reviewed, highlighting how these compounds respond to both AC and DC excitations, which is particularly relevant for applications involving colloidal stability and transport optimization.^4^ Another fundamental aspect is the role of physicochemical conditions, such as temperature and pressure, which affect asphaltene deposition during the production and transportation stages of petroleum. Recent studies have shown that under high-temperature and high-pressure conditions, the tendency of asphaltene precipitation increases, potentially compromising reservoir operations.^5^ In addition, the interaction between asphaltenes and wax has been investigated, revealing that the aggregation state of asphaltenes, whether dispersed or aggregated, strongly influences wax deposition.^6^

From a methodological standpoint, there has also been significant progress in the use of EIS for nonideal systems. Recent reviews emphasize that many electrochemical processes are not strictly linear nor stationary, which can compromise the traditional interpretation of impedance spectra.^7^ Moreover, modern approaches have demonstrated how to distinguish different contributions within impedance data—such as charge-transfer resistance, diffusion, and interfacial effects—applicable to both electrochemical materials and complex organic systems.^8^ Against this background, the present work advances the dielectric characterization of crude oils and their fractions using EIS over a wide frequency range, combining equivalent-circuit analysis with rigorous model validation. The objective is to elucidate the mechanisms of conduction, relaxation, and structural heterogeneity across different samples, thereby contributing to a deeper understanding of asphaltene physics and supporting industrial applications related to oil stability, aggregation, and transport.

## 2 Results and Discussion

### 2.1 Oil Chemical Composition: Saturated and Aromatic Hydrocarbons, Resins, and Asphaltenes

The results regarding the percentages of saturated and aromatic hydrocarbon fractions, resins, which make up the maltenic fraction, and asphaltenes of the two oil samples are presented in Table 1. This table also presents the results from the precipitation of asphaltenes from the oils studied.

**Table 1:** Composition in % (Weight) of Saturated and Aromatic Hydrocarbons, Resin + Asphaltene Fractions, and the Percentages of Asphaltene Precipitated from M and A Oil Samples

oil sample	saturated (%)	aromatics (%)	resins + asphaltenes (%)	asphaltene precipitated
A	33.83	27.90	25.93	1.38
M	28.50	24.00	36.75	5.33

In evaluating the M Oil sample, it was possible to verify that this oil has a lower percentage of saturated hydrocarbons (28.50%) and aromatics (24.00%) when compared to A Oil, (33.83%) and (27.90%), respectively. This difference is a consequence of the microbial activity on M Oil, which occurred within its reservoir. In this process, microorganisms degrade saturated *n*-alkanes and branched hydrocarbons, followed by low-molecular weight aromatic hydrocarbons, high-molecular aromatic hydrocarbon and resins, preferentially, with asphaltenes being the last to be biodegraded.^9–11^ Consequently, the heavier and more polar fractions (resins and asphaltenes), which are more difficult to biodegrade, end up concentrating in the oil. This explains why M Oil has more resins + asphaltenes (36.75%), when compared to A Oil (25.93%) and, mainly, a much higher percentage of asphaltenes (5.33% in M Oil and 1.38% in A Oil).

### 2.2 Analysis of Nyquist Diagrams

The Nyquist diagrams (Figure 1) exhibit a single semicircular arc for all samples, a characteristic feature of highly resistive dielectric systems in which the response is dominated by a low-frequency dielectric relaxation process.^12^ The diameter of this semicircle along the real axis (*Z*′) is directly proportional to the charge-transfer resistance (Rct), which, in the context of crude oils, represents the overall resistance to the mobility of charged species (either ionic or dipolar) as well as the degree of medium polarization under an applied electric field.^13^

**Figure 1:**
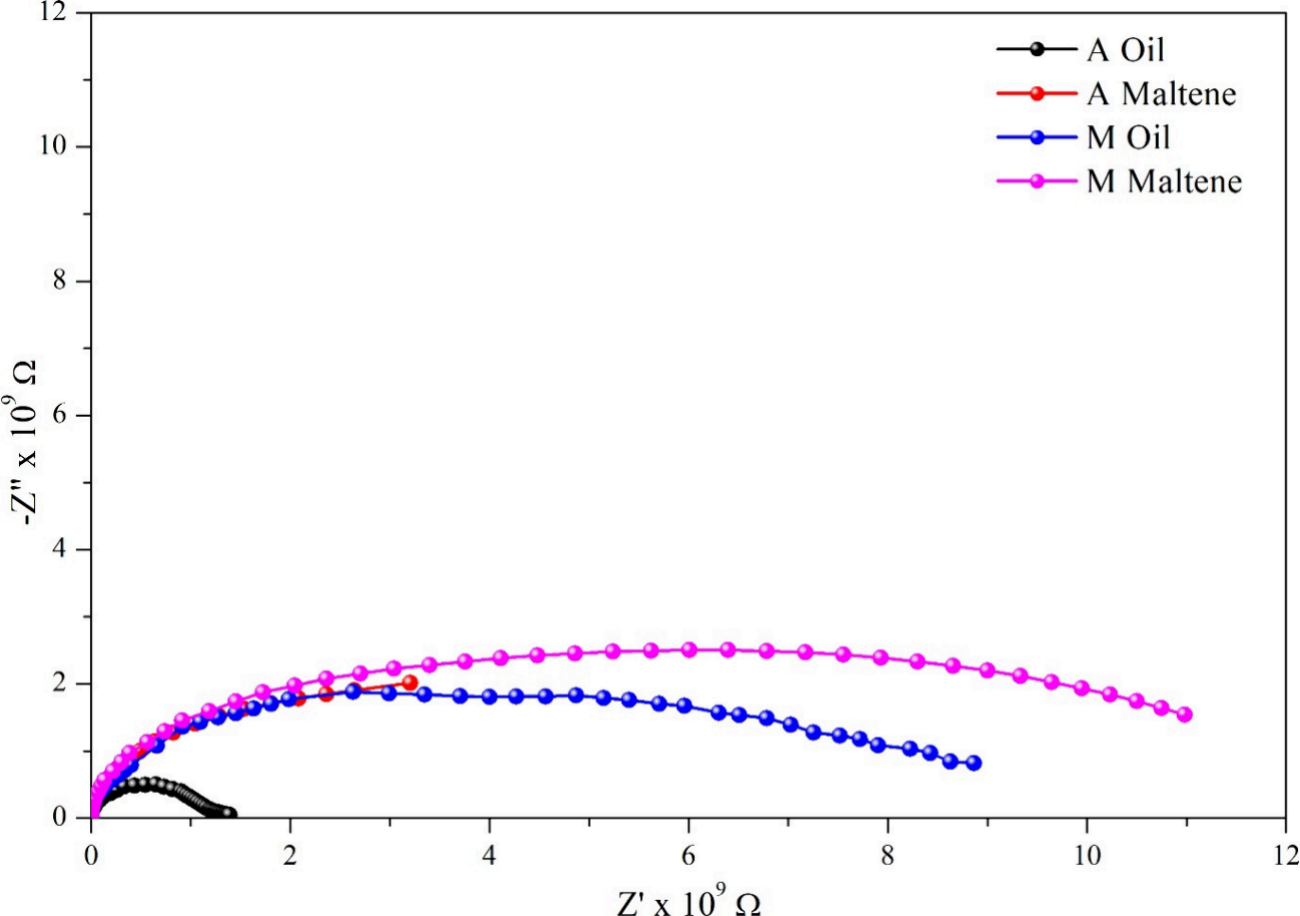
Nyquist plots for the total oil samples (A Oil and M Oil) and their corresponding maltenes (A Maltene and M Maltene).

A visual inspection of Figure 1 clearly establishes the resistivity order: Rct (A Oil) < Rct (A Maltene) < Rct (M Oil) < Rct (M Maltene). The M Oil sample and M Maltene exhibit substantially higher Rct values, indicating that both the crude oil and its corresponding maltene fraction possess a more insulating molecular matrix with greater dielectric stability compared with those from the A Oil sample. This difference reflects the underlying chemical composition and molecular-weight distribution, with the M Oil being characteristic of a heavier and structurally more complex crude oil.^14^ The resin and asphaltene fractions of M Oil are more enriched in organic compounds containing heteroatoms of nitrogen, sulfur, and oxygen when compared to the same fractions of A Oil.

The Bode plots (Figure 2) further support this conclusion. The impedance magnitude (|*Z*|) versus frequency (Figure 2A) shows that the M Oil sample retains substantially higher impedance values at low frequency, consistent with their higher DC resistivity. The phase angle (θ) response as a function of frequency (Figure 2B) reveals that all samples exhibit predominantly capacitive behavior at high frequencies (θ → −90°), as expected for dielectric media. The depression of the phase peak and the gradual slope in the low frequency region provide critical insight into the degree of microstructural heterogeneity within each sample.

**Figure 2:**
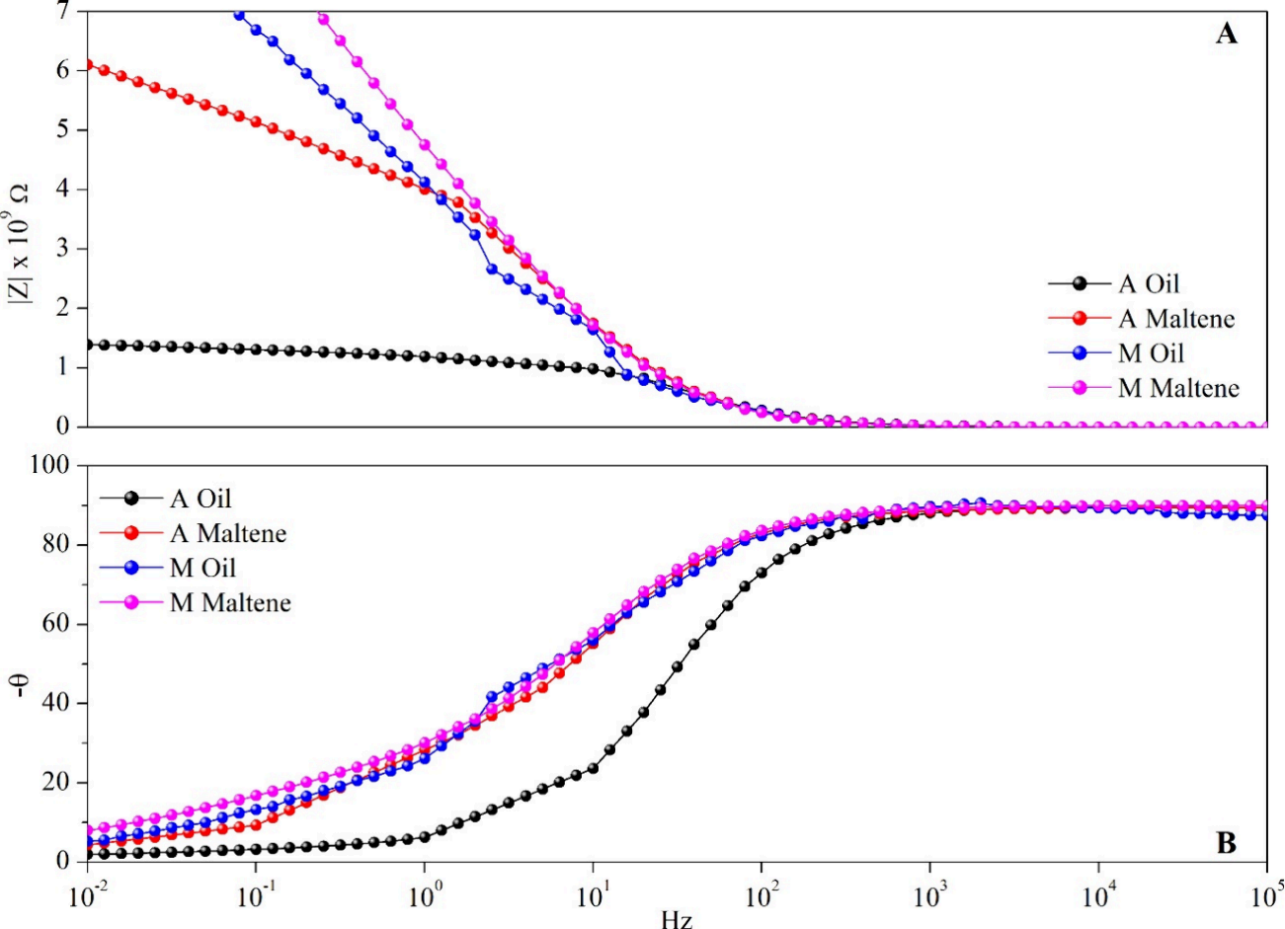
Bode plots: (A) impedance magnitude (|*Z*|) as a function of frequency; (B) phase angle (θ) as a function of frequency.

### 2.3 Equivalent Circuit Modeling and Physical Interpretation

To quantitatively describe the relaxation and conduction processes, the impedance data were fitted using Equivalent Electrical Circuits (EECs). The depression of the Nyquist semicircles, more pronounced for the M Oil samples, requires replacing the ideal capacitor with a Constant Phase Element (CPE), which is indicative of frequency dispersion arising from physical or chemical heterogeneity within the system.^15^ Table 2 summarizes the EEC models used for data fitting as well as the results of the Kramers–Kronig (KK) validation analysis.

**Table 2:** Kramers–Kronig Validation of the Impedance Spectra for the Proposed Fitted Circuits

		Kramers–Kronig		
sample	circuit	χ^2^ (*Z*)	χ^2^ (*Z*′)	χ^2^ (−*Z*″)
A Oil	C_1_[R_1_(R_2_ CPE_1_)]	8.09 × 10^–10^	3.39 × 10^–10^	4.69 × 10^–10^
A Maltene	CPE_1_(R_1_ CPE_2_)	1.07 × 10^–8^	4.69 × 10^–8^	6.00 × 10^–8^
M Oil	R_1_(R_2_ CPE_1_) CPE_2_	3.50 × 10^–9^	1.56 × 10^–9^	1.94 × 10^–9^
M Maltene	C_1_[R_1_(R_2_ CPE_1_)]	5.99 × 10^–9^	2.91 × 10^–9^	3.07 × 10^–9^

The selection of the equivalent electrical circuits was based not only on the quality of the fitting but also on the physical interpretation of the dielectric response of each system. Crude oils and maltene fractions are inherently heterogeneous media composed of molecules with different polarities, aggregation states, and intermolecular interactions, which give rise to nonideal relaxation behavior. Consequently, ideal capacitive elements were replaced by Constant Phase Elements (CPEs) whenever required to account for the distribution of relaxation times associated with structural heterogeneity, molecular aggregation, and interfacial polarization phenomena.

For each sample, the simplest circuit capable of accurately reproducing the experimental impedance spectra was selected. The resistive elements (R_1_ and R_2_) were associated with distinct contributions to bulk electrical conduction and dielectric losses within the oil matrix, whereas the capacitive and CPE elements describe polarization processes arising from molecular organization and local variations in electrical properties. Additional circuit elements were introduced only when necessary to reproduce specific spectral features observed in the Nyquist and Bode plots. The validity of the selected models is supported by the excellent agreement between experimental and fitted spectra, as well as by the successful Kramers–Kronig validation and the very low χ^2^ values obtained for all samples.

The low magnitude of the χ^2^ values for all samples confirms the physical reliability and internal consistency of the EIS data, a fundamental requirement for meaningful interpretation of the extracted circuit parameters. In addition, Table 3 reports the quantitative parameters obtained from fitting each sample to its corresponding EEC model. Furthermore, the differences observed among the selected equivalent circuits reflect variations in the structural organization and dielectric heterogeneity of the investigated systems, indicating that the complexity of the adopted models is consistent with the compositional differences between crude oils and their corresponding maltene fractions.

**Table 3:** Equivalent Circuit Parameters Obtained from EIS Fitting for Crude Oils and Maltene Fractions

		CPE_1_		CPE_2_			
sample	R_1_ (Ω)	Y_0_ (S s^*n*^)	*n*	Y_0_ (S s^*n*^)	*n*	C_1_ (pF)	R_2_ (Ω)
A Oil	8.49 × 10^8^ (±0.38%)	5.89 × 10^–10^ (±2.48%)	0.427 (±1.74%)			5.39 (±0.29%)	5.78 × 10^8^ (±1.17%)
A Maltene	1.69 × 10^9^ (±4.50%)	6.32 × 10^–12^ (±0.63%)	0.994 (±0.06%)	1.67 × 10^–10^ (±4.60%)	0.349 (±3.75%)		
M Oil	7.95 × 10^8^ (±4.71%)	6.32 × 10^–12^ (±0.89%)	0.998 (±0.09%)	7.67 × 10^–11^ (±2.37%)	0.461 (±2.31%)		8.75 × 10^9^ (±1.83%)
M Maltene	1.71 × 10^9^ (±2.82%)			9.04 × 10^–11^ (±1.83%)	0.451 (±1.75%)	6.35 × 10^–12^ (±0.60%)	1.17 × 10^10^ (±1.63%)

The Nyquist plot for A Oil (Figure 3) exhibits the smallest semicircle diameter among the four samples, indicating the lowest charge-transfer resistance (Rct). This parameter reflects the overall resistance to the mobility of charged species, whether ionic, dipolar, or supramolecular aggregates within the fluid medium.^16^ The equivalent circuit employed, C_1_[R_1_(R_2_ CPE_1_)], suggests the presence of a dominant relaxation process. The combination of an ideal capacitor (C_1_) with the parallel (R_2_ CPE_1_) branch suggests the coexistence of bulk dielectric polarization and nonideal interfacial polarization phenomena, the latter being represented by the CPE element.^17^ The relatively low Rct obtained for A Oil indicates that this sample possesses a less restrictive structural matrix for charge motion, an attribute typically associated with lighter, less chemically complex, or more mature oils. Oil A has a lower percentage of resin and asphaltene (25.93%) when compared to M Oil (36.75%). This behavior contrasts with that of M Oil, whose higher Rct implies a denser or more heterogeneous medium that imposes greater hindrance to charge mobility.^14^

**Figure 3:**
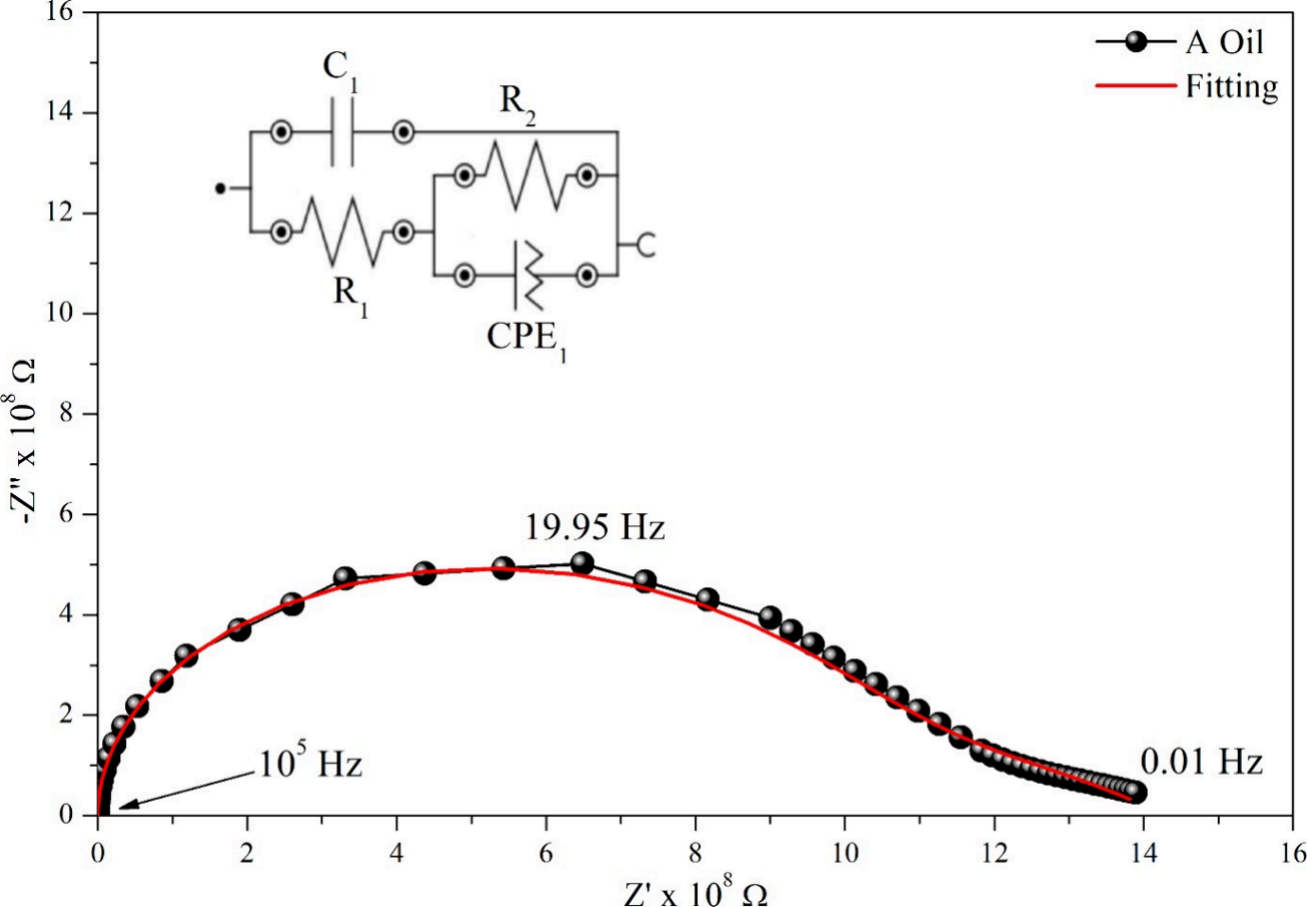
Nyquist plot of the A Oil sample showing the experimental impedance data (markers) and the corresponding fit obtained using the equivalent circuit model (red line). The inset illustrates the equivalent circuit employed, composed of R_1_, C_1_, R_2_, and CPE_1_. Characteristic frequencies along the semicircle are indicated.

The Nyquist plot for A Maltene (Figure 4) shows a semicircle with a larger diameter than that of A Oil, resulting in a higher charge-transfer resistance, Rct (≈ 3.0 × 10^9^ Ω). This is a particularly important result, as it demonstrates that removing asphaltenes, the most polar fraction of crude oil, yields a material with substantially increased insulating behavior. The equivalent circuit used, CPE_1_(R_1_ CPE_2_), reflects a broader distribution of dielectric relaxation processes, consistent with the increased heterogeneity of the maltene matrix after asphaltene removal. The presence of two nonideal capacitive elements suggests that polarization phenomena occur over a wider range of characteristic times, indicating a more distributed dielectric response.^18^

**Figure 4:**
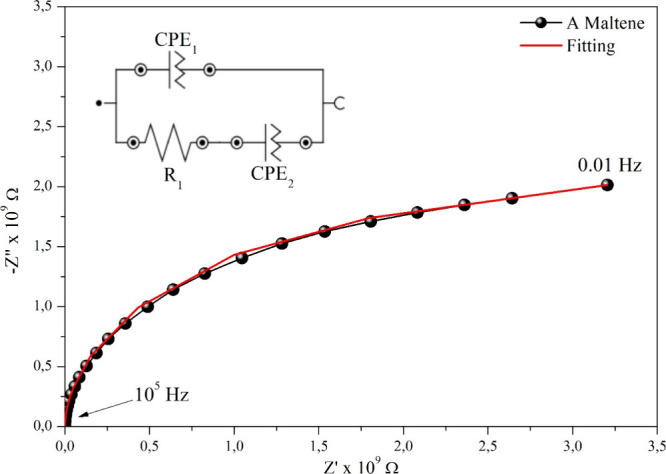
Nyquist plot of the A Maltene sample showing the experimental impedance data (markers) and the corresponding fit obtained using the equivalent circuit model (red line). The inset illustrates the equivalent circuit employed, composed of R_1_, CPE_1_, and CPE_2_. Characteristic frequencies along the semicircle are indicated.

The transition from the simpler circuit required for A Oil to the more distributed model necessary for A Maltene, combined with the marked increase in resistivity, reinforces the idea that asphaltenes, even at low percentage (1.38%, Table 1), act as charge-active centers, facilitating conduction in crude oil through mechanisms such as charge hopping.^14^ Once removed, the isolated maltene matrix behaves as a structurally more uniform and significantly more efficient dielectric.

As evidenced in Figure 5, the Nyquist plot of M Oil exhibits a substantially broader semicircle compared with A Oil and A Maltene, corresponding to an extremely high charge-transfer resistance of approximately Rct ≈ 8.5 × 10^9^ Ω. Such a large Rct strongly suggests that M Oil is a denser crude, possessing a more insulating and structurally organized molecular matrix—features typically associated with heavy oils that form stable supramolecular aggregates. Recent molecular-simulation studies have shown that asphaltene aggregation in systems with high aromaticity and heteroatom content results in dense and persistent supramolecular structures, thereby hindering charge mobility.^19^

**Figure 5:**
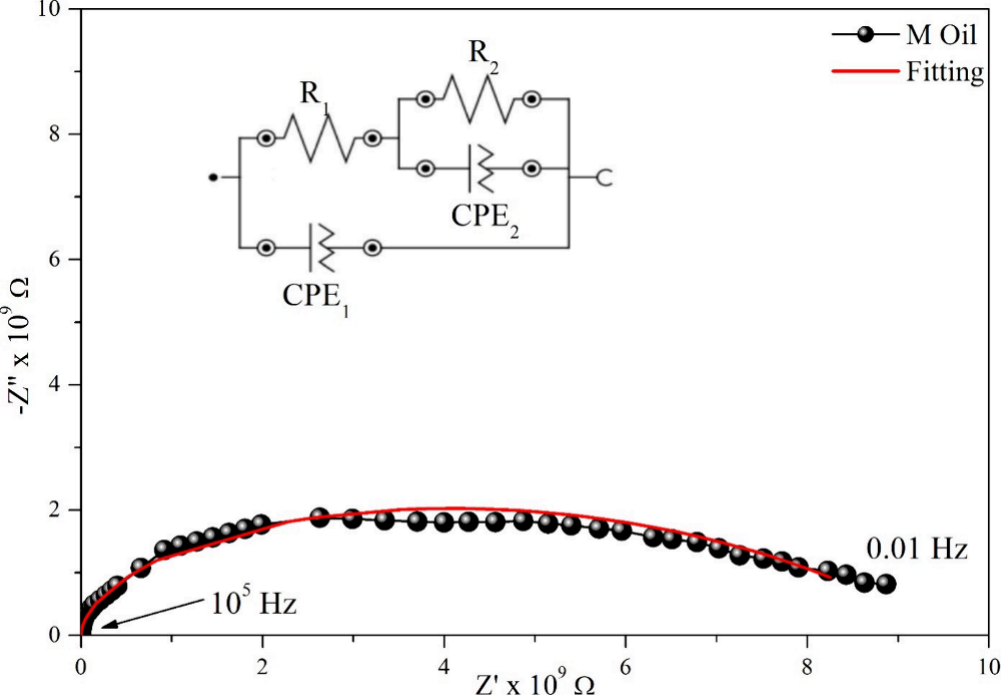
Nyquist plot of the M Oil sample showing the experimental impedance data (black dots) and the corresponding fit obtained using the equivalent circuit model (red line). The inset illustrates the equivalent circuit employed, composed of R_1_ in series with CPE_1_, and this combination in series with a parallel combination of R_2_ and CPE_2_. Characteristic frequencies along the semicircle are indicated.

The equivalent-circuit model using the R_1_(R_2_ CPE_1_) CPE_2_ topology further reinforces this interpretation. This configuration indicates a broader distribution of relaxation phenomena and enhanced microstructural heterogeneity, likely arising from the coexistence of asphaltene-rich domains and other oil constituents with different dielectric properties. Such behavior is consistent with recent reports describing the aggregation and disaggregation dynamics of asphaltenes in response to changes in solvency and phase composition.^20^ Taken together, the exceptionally large Rct value and the complexity of the equivalent-circuit model indicate the formation of organized supramolecular domains that actively restrict charge transport. This mechanism is particularly relevant in heavier crudes, where asphaltenes tend to self-associate into stable nanoaggregates, as supported by simulations demonstrating that the molecular polydispersity of asphaltenes promotes the stabilization of these domains.^19^

As shown in Figure 6, the Nyquist diagram of M Maltene displays the largest semicircle diameter among all samples, corresponding to a maximum charge-transfer resistance of approximately Rct ≈ 1.1 × 10^10^ Ω. This result reinforces the trend observed for the A pair: the removal of asphaltenes substantially increases the insulating character of the maltene matrix. Interestingly, the equivalent circuit that best fits the M Maltene data reverts to the simpler topology C_1_[R_1_(R_2_ CPE_1_)], the same model used for A Oil, but with drastically different resistance and CPE parameters. The exceptionally high Rct confirms that M Maltene behaves as the most effective dielectric medium among all samples, exhibiting the lowest charge-carrier mobility. The simplification of the relaxation behavior, now dominated by a single relaxation contribution, suggests that the removal of asphaltenes not only increases resistivity but also promotes a more homogeneous dielectric response. In other words, eliminating asphaltene aggregates suppresses one of the relaxation pathways originally present in the full M Oil sample.

**Figure 6:**
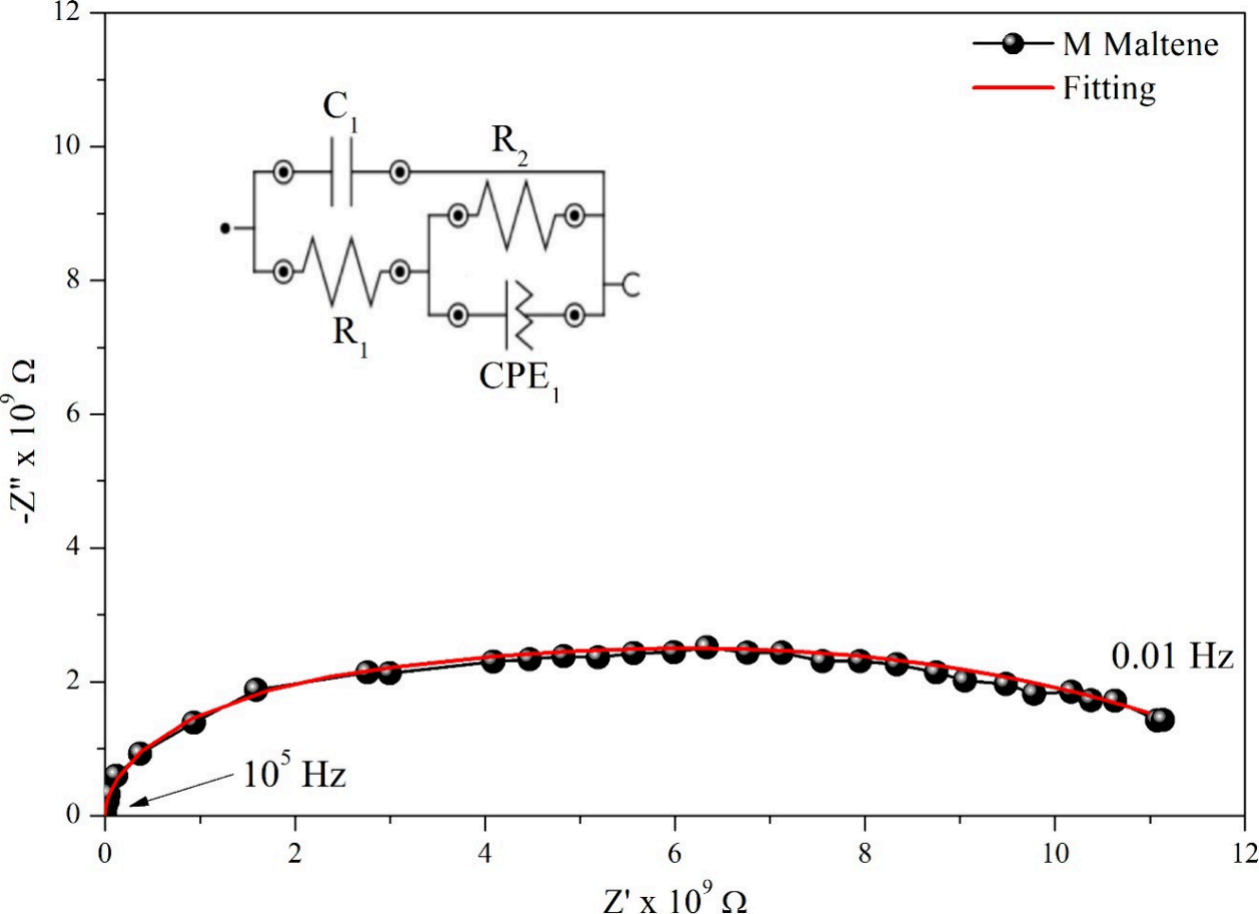
Nyquist plot of the M Maltene sample showing the experimental impedance data (black dots) and the corresponding fit obtained using the equivalent circuit model (red line). The inset illustrates the equivalent circuit employed, composed of R_1_ in parallel with C_1_, and this combination in series with the parallel combination of R_2_ and CPE_1_. Characteristic frequencies along the semicircle are indicated.

These observations are consistent with recent research. Pétuya et al.^21^ showed through molecular dynamics simulations and machine-learning analysis that molecular polydispersity strongly influences asphaltene aggregation behavior, ultimately affecting macroscopic properties such as charge transport. Ma et al.^22^ demonstrated that solvent quality modulates the aggregation state of asphaltenes at oil–water interfaces, altering film rigidity and electrical response. Furthermore, Peng et al.^19^ reported that asphaltene aggregates undergo dynamic structural reorganization under shear, influencing supramolecular stability, an insight that aligns with our interpretation of enhanced dielectric rigidity following asphaltene removal.

The resistivity hierarchy observed (Rct (A Oil) < Rct (A Maltene) < Rct (M Oil) < Rct (M Maltene)) reveals that both geological origin and the presence of asphaltenes strongly influence the dielectric behavior of these systems. The consistently higher resistivity of samples from M origin (M Oil and M Maltene) indicates that this crude is intrinsically heavier and structurally more complex. Oils with higher molecular weights, increased branching, and greater heteroatom content tend to exhibit stronger intermolecular interactions, such as π–π stacking and hydrogen bonding, leading to the formation of more stable supramolecular domains that restrict charge mobility and increase overall impedance. In addition, the possible influence of trace amounts of water cannot be completely excluded, since water molecules may participate in hydrogen-bonding networks and affect the organization of supramolecular domains. However, the present study did not include direct moisture-content measurements, and therefore no specific contribution of water to the observed impedance response can be established. Consequently, the interpretations presented herein are primarily based on the compositional differences between crude oils and their corresponding maltene fractions, particularly regarding the role of asphaltenes and molecular aggregation phenomena.

This trend aligns well with recent insights into the microstructure and electronic behavior of heavy crude components. Moreover, the finding that the maltene fractions consistently show higher Rct values than their corresponding whole oils (i.e., Rct (Maltene) > Rct (Oil)) challenges the traditional view of maltenes as merely an inert solvent. Recent studies have shown that asphaltenes can act as active charge-transport centers. Both experimental and computational investigations demonstrate that asphaltene aggregates can create conductive pathways via charge hopping or aromatic–domain–assisted transport.^23,24^ Once the asphaltenes are removed, the isolated maltene matrix behaves as a purer and more homogeneous dielectric medium, devoid of these active centers, thus exhibiting substantially higher resistivity. This interpretation is consistent with recent dielectric spectroscopy analyses and molecular modeling studies showing that asphaltenes modulate relaxation and conduction processes in complex hydrocarbons.^25^

An additional aspect that deserves consideration is the potential influence of the applied AC electric field on the supramolecular organization of crude-oil constituents during impedance measurements. In complex petroleum systems containing polar fractions such as asphaltenes, electric-field-induced molecular orientation, aggregate rearrangement, or polarization effects may occur, particularly at low frequencies where the applied field acts over longer time scales. Although no evidence of measurement instability or progressive spectral changes was observed under the experimental conditions employed in this study, the possibility that the measurement itself may induce subtle structural perturbations cannot be completely excluded. Further investigations involving field-amplitude-dependent EIS measurements and time-resolved studies would be valuable to clarify the extent of such effects in crude-oil systems.

Another aspect that may provide further insight into the conduction and polarization mechanisms of crude oils and maltene fractions is the temperature dependence of the impedance response. Variations in temperature can influence molecular mobility, intermolecular interactions, aggregate stability, and charge-transport processes, thereby affecting the dielectric properties of these complex systems. Although the present study was conducted under controlled laboratory conditions at a fixed temperature, future temperature-dependent EIS investigations could provide valuable information regarding activation processes and the role of supramolecular organization in charge transport.

Taken together, the resistivity order observed in this work reflects both (i) the intrinsic structural differences between light and heavy crudes and (ii) the supramolecular role of asphaltenes in facilitating charge mobility. These two factors jointly dictate the dielectric response and impedance characteristics of the fractions analyzed, in agreement with the most recent developments reported in the literature.

## 3 Conclusions

The application of EIS for the dielectric characterization of crude oils and their maltene fractions has proven to be a powerful tool for investigating complex organic systems. The results reveal that the presence of asphaltenes and the geological origin of the crude oil are determining factors in their dielectric properties and charge transport. A clear resistivity hierarchy was established (Rct (A Oil) < Rct (A Maltene) < Rct (M Oil) < Rct (M Maltene)), indicating that M Oil is intrinsically heavier and more complex than A Oil. Systematic analysis showed that asphaltenes act as charge-active centers, facilitating conduction through mechanisms such as charge hopping or supramolecular pathways. Their removal results in a substantial increase in the insulating character of the maltene matrix, significantly raising the charge-transfer resistance. Equivalent circuit modeling revealed that asphaltene removal not only increases resistivity but also homogenizes the dielectric response by suppressing relaxation pathways, as observed in the transition from M Oil to M Maltene. These findings provide experimental validation of the role of asphaltene aggregation in modulating electrical properties and suggest that EIS can serve as a valuable diagnostic tool for monitoring asphaltene behavior and crude oil stability in industrial applications.

## 4 Experimental Section

### 4.1 Crude Oil Samples

The crude oil samples, A and M, used in this study were obtained from Campos Basin offshore oil fields. This basin is located in the northeastern of Rio de Janeiro, Brazil. These fields are situated in average water depths of approximately 800 m.^26,27^ The oils accumulated in the Campos Basin reservoirs are derived from its source rock, Lagoa Feia Formation, sedimentary rock deposited during the separation of the South American and African continents in the Cretaceous period.^28^ Consequently, the M and A oils share a common geochemical origin associated with the same source rock,^29^ minimizing compositional variability. Thus, the samples differ mainly in relevant physicochemical properties, such as API gravity and biodegradation level. The samples were supplied by Petrobras, and API gravity measurements were carried out at the Petroleum Engineering and Exploration Laboratory. The A Oil exhibited an API gravity of 29.0°, whereas the M Oil presented an API gravity of 19°.

### 4.2 Asphaltene Precipitation

Approximately 150 mg of both crude oil samples were weighed into 15 mL glass tubes. Subsequently, 50 μL of dichloromethane and 6 mL of *n*-hexane were added to disrupt the colloidal stability of the resin–asphaltene aggregates, present in the sample, and asphaltene precipitation. The mixtures were sonicated for 5 min to promote the disaggregation of resin–asphaltene complexes and then centrifuged at 3000 rpm for 5 min. After centrifugation, the supernatant containing the maltene fraction (saturated and aromatic hydrocarbons, and resins) was carefully removed using a Pasteur pipet and transferred to a beaker. The procedure, addition of 50 μL of dichloromethane and 6 mL of *n*-hexane, followed by sonication, centrifugation, and removal of the supernatant, was repeated three additional times to ensure complete separation of the remaining maltenes from the asphaltene fraction.

### 4.3 Separation of the Components of the Maltenic Fraction

The maltenic fraction, obtained from the asphaltene precipitation of the two oils, was subjected to chromatographic fractionation in an open column for the separation of saturated and aromatic hydrocarbons, and resins. Approximately 40 mg of this fraction, adsorbed on alumina, were applied to a glass chromatographic column containing 5 g of silica gel (Merck, 230–400 mesh) and *n*-hexane. For the elution of saturated hydrocarbons, 30 mL of *n*-hexane was used; for the elution of aromatic hydrocarbons, 30 mL of a mixture of *n*-hexane and dichloromethane (8:2) was used; and for the resins, 30 mL of a mixture of dichloromethane and methanol (9:1) was used. All fractions were collected in previously weighed glass beakers, and after complete evaporation of the solvents, the beakers were weighed again to calculate the gravimetric mass (%) of each fraction obtained. The corresponding maltene fractions are identified as Maltene A (from oil sample A) and Maltene M (from oil sample M). These labels are used consistently throughout the manuscript to ensure clarity in sample comparison.

### 4.4 Electrochemical Measurements

The crude oil samples and maltene fractions were analyzed by Electrochemical Impedance Spectroscopy (EIS) at 25 ± 0.1 °C. Owing to the high impedance of the samples, the electrochemical cell consisted of two identical rectangular carbon-steel electrodes separated by a 5 mm gap, defining an effective volume of approximately 10 cm^3^. Similar configurations have been employed for the characterization of ceramics and polymeric materials.^30^ All measurements were conducted inside a Faraday cage to minimize external electromagnetic interference. The system was required to satisfy the principles of linearity, stationarity, and causality, and its validity was confirmed through Kramers–Kronig (KK) transformations. For each sample, the average time to reach steady-state potential and current was approximately 3600 s. Impedance spectra were acquired over the frequency range of 10 kHz to 10 mHz with a logarithmic distribution of 10 points per decade, using an AC perturbation amplitude of 200 mV. All experiments were performed with an AUTOLAB PGSTAT128N potentiostat/galvanostat equipped with an FRA32 module. To assess measurement reproducibility, three independent EIS measurements were performed for each sample under identical experimental conditions. The resulting impedance spectra exhibited excellent overlap and no significant variations were observed among replicate measurements. Variations in the fitted equivalent-circuit parameters were typically below 5%, confirming the robustness and reproducibility of the experimental procedure.
